# Improvement in the biochemical and chemical properties of badland soils by thorny bamboo

**DOI:** 10.1038/srep40561

**Published:** 2017-01-19

**Authors:** Yo-Jin Shiau, Hsueh-Ching Wang, Tsai-Huei Chen, Shih-Hau Jien, Guanglong Tian, Chih-Yu Chiu

**Affiliations:** 1 Biodiversity Research Center, Academia Sinica, Taipei 11529, Taiwan; 2 Taiwan Forestry Research Institute, Taipei 10066, Taiwan; 3 Department of Soil and Water Conservation, National Pingtung University of Science and Technology, Pingtung 912-01, Taiwan; 4 Environmental Monitoring and Research Division, Monitoring and Research Department, Metropolitan Water Reclamation District of Greater Chicago, 6001 W. Pershing Road, Cicero, IL 60804, USA

## Abstract

Badland soils—which have high silt and clay contents, bulk density, and soil electric conductivity— cover a large area of Southern Taiwan. This study evaluated the amelioration of these poor soils by thorny bamboo, one of the few plant species that grows in badland soils. Soil physiochemical and biological parameters were measured from three thorny bamboo plantations and nearby bare lands. Results show that bamboo increased microbial C and N, soil acid-hydrolysable C, recalcitrant C, and soluble organic C of badland soils. High microbial biomass C to total organic C ratio indicates that soil organic matter was used more efficiently by microbes colonizing bamboo plantations than in bare land soils. High microbial respiration to biomass C ratio in bare land soils confirmed environmentally induced stress. Soil microbes in bare land soils also faced soil organic matter with the high ratio of recalcitrant C to total organic C. The high soil acid-hydrolysable C to total organic C ratio at bamboo plantations supported the hypothesis that decomposition of bamboo litter increased soil C in labile fractions. Overall, thorny bamboo improved soil quality, thus, this study demonstrate**s** that planting thorny bamboo is a successful practice for the amelioration of badland soils.

The badland soil that is derived from mudrock (a class of fine-grained siliciclastic sedimentary rock) is unfavourable for plant growth because of its high clay and calcium carbonate contents^1^. One of the common physiochemical properties of the badland soil is the low water infiltration, thus, most rainfalls on badland fields drain via surface runoff, leading to soil erosion and nutrient loss^2^. Moreover, in badland soils, sodium and chlorine have been found to concentrate near soil surface during the dry season, which creates repulsive forces among soil particles, causing the soil to become rock-hard. On the other hand, during the rainy season, soils swell and soften upon water saturation, which has been shown to accelerate surface erosion^3^. Concentrated sodium and chlorine in this type of soil also increase soil electric conductivity, rendering it inhospitable to plant growth, resulting in bare landscapes in most badland ecosystems.

In Southern Taiwan, Plio-Pleistocene badland soils, consisting of up to 90% silt and clay combined, occupy more than 10,000 ha and are devoid of vegetation^1^. Soil crusting is common on the surface of this badland soil, which increases its bulk density and penetration resistance. Previous research^4^ has indicated that root growth could be inhibited when penetration resistance exceeds 14 kg cm^−2^, where the penetration resistance of the bare soil in the badlands of Southern Taiwan has been shown to be high (14.5 ± 1.47 kg cm^−2^)^5^.

Thorny bamboo (*Bambusa stenostachya*), a dense clumping bamboo with an average height of 12–15 m, is one of the only plant species that are able to grow in these uninhabitable soils. Research has shown that bamboo plants absorb nutrients from deep soils and retain soil water in infertile environments, as they are known to have deeper rhizomes and roots than many other non-woody plants^6^. A previous study reported that the ecological parameters of a highly degraded soil in India were significantly improved as a result of the increase in soil organic matter (SOM) by planting bamboos^7^.

Soil organic matter consists of different C fractions such as soluble, acid hydrolysable and recalcitrant C. In addition to the overall SOM pools increased by bamboo plantations, humification and the composition of SOM may be a good reflection of the impact of the plant on SOM^8^. During humification, organic compounds in plant litter could be transformed into humic substances, whereupon their structures become significantly altered compared to the original forms in plant materials^9^. To the best of our knowledge, the mechanisms by which thorny bamboo grows in badland ecosystems, and the interactions between the growth of the bamboo, soil nutrients, and microorganisms in this hostile soil environment are still not well understood. Thus, the objective of the study was to find out the difference in physical, chemical, and biological parameters of soil between bamboo plantations and adjacent bare lands in Southern Taiwan. We hypothesized that the presence of bamboo in these soils would result in significant increases in SOM and soil nutrients, and that the increase in SOM would improve soil physiochemical properties and increase soil microbial biomass.

## Results

The analysis of soil properties from the three sampling locations is shown in Table 1. Soils were mostly loam to clay loam, containing high silt and/or clay content. Soil was slightly alkaline (pH = 8.3) at all locations except at the bamboo plantation in Site 2, where the soil was slightly acidic (pH = 6.2). Generally, the soil was distinctively different between bamboo plantations and bare land soils. Soil electric conductivity was significantly lower in bamboo plantation soils (0.7 mS cm^−1^) than in bare land soils (11.2 mS cm^−1^) (p < 0.05), and soil water content was higher at bamboo plantations than in bare land soils (p < 0.05).

The soluble organic carbon (S_b_OC) content, measured using potassium chloride (KCl) extracts (S_b_OC_KCl_), was much higher at bamboo plantations than in bare land soils (p < 0.05) (Table 2). Site 2 had the highest S_b_OC_KCl_ among the bamboo plantations, which was more than 2 times higher compared to bare land soil (p < 0.05). A similar trend was observed for S_b_OC using the hot water extraction method (S_b_OC_HW_). The mean S_b_OC_HW_ was nearly 6 times higher in bamboo plantation soils than bare land soils (p < 0.05), while the highest S_b_OC_HW_ was measured in the bamboo plantation soil from Site 2 (Table 3). The S_b_OC values from all locations were higher from samples extracted using the hot water method compared to samples extracted using KCl. The bare land soils from the three sampling locations had similar levels of S_b_OC, irrespective of the extraction method used.

The difference in extractable nitrogen (N) between bamboo plantations and bare land soils depended on N species. The concentrations of KCl-extracted NH_4_^+^ (NH_4_^+^_KCl_) and total dissolved nitrogen (TDN_KCl_) at the three sites were 2–4 times higher at bamboo plantations than in bare land soils (p < 0.05), whereas, KCl-extracted NO_3_^−^ (NO_3_^−^_KCl_) and soluble organic nitrogen (S_b_ON_KCl_) were similar between bamboo plantations and bare land soils. Similarly, the concentration of NH_4_^+^_HW_ and TDN_HW_ were 3–10 times higher in bamboo plantation soils than in bare land soils (p < 0.05), whereas NO_3_^−^_HW_ and S_b_ON_HW_ were not significantly different between bamboo plantations and bare land soils, with the exception of S_b_ON_HW_ from Site 2, where NO_3_^−^_HW_ and S_b_ON_HW_ were higher in bamboo plantation soils than bare land soils. The S_b_OC_HW_ to total organic carbon (TOC) ratio (S_b_OC_HW_/TOC) was found to be higher in bamboo plantation soils than in bare land soils, however, the opposite trend was observed for S_b_OC_KCl_/TOC. No differences in the S_b_ON_HW_ or S_b_ON_KCl_ to total nitrogen (TN) ratios (S_b_ON_HW_/TN or S_b_ON_KCl_/TN) were observed between bamboo plantations and bare land soils.

Soil microbial biomass C (C_mic_) and N (N_mic_) were found to be 7–10 times higher in bamboo plantation soils than in bare land soils (p < 0.05) (Table 4). Soil respiration rates and potentially mineralisable N were also higher at bamboo plantations than in bare land soils. The amount of total mineralisable N was almost negligible in bare land soils, although a slight respiration rate was detected. The C_mic_/TOC and N_mic_/TN ratios were higher at bamboo plantations than in bare land soils (p < 0.05), whereas respiration/C_mic_ was higher in bare land soils than in bamboo plantation soils (p < 0.05).

**Figure 1:**
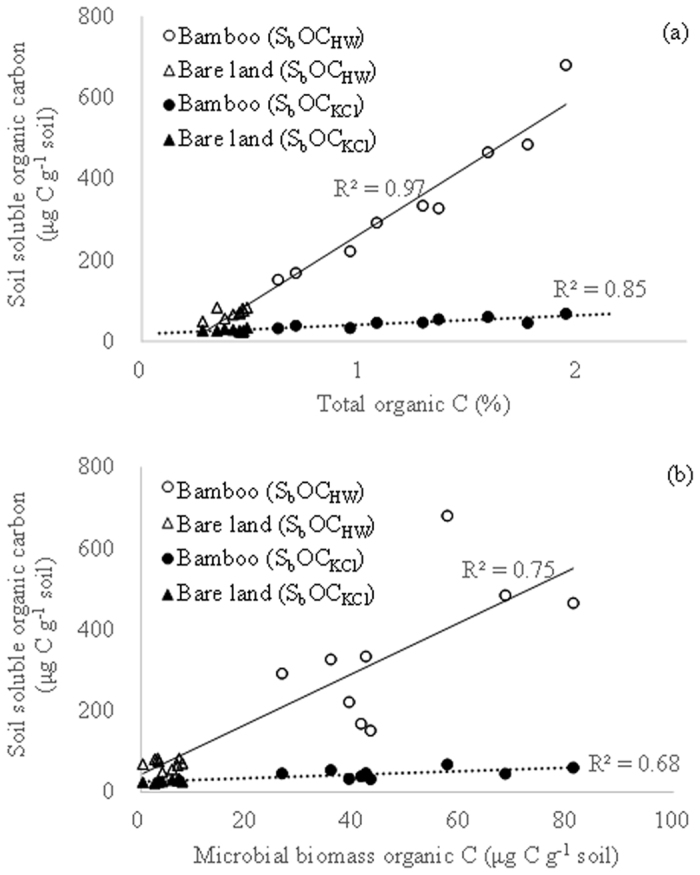
Relationship between soil soluble organic carbon in 2 M KCl extracts (S_b_OC_KCl_) and soil total organic carbon (**a**) and that between hot water extracts (S_b_OC_HW_) and soil microbial biomass carbon (**b**) in thorny bamboo plantations and adjacent bare lands.

**Figure 2:**
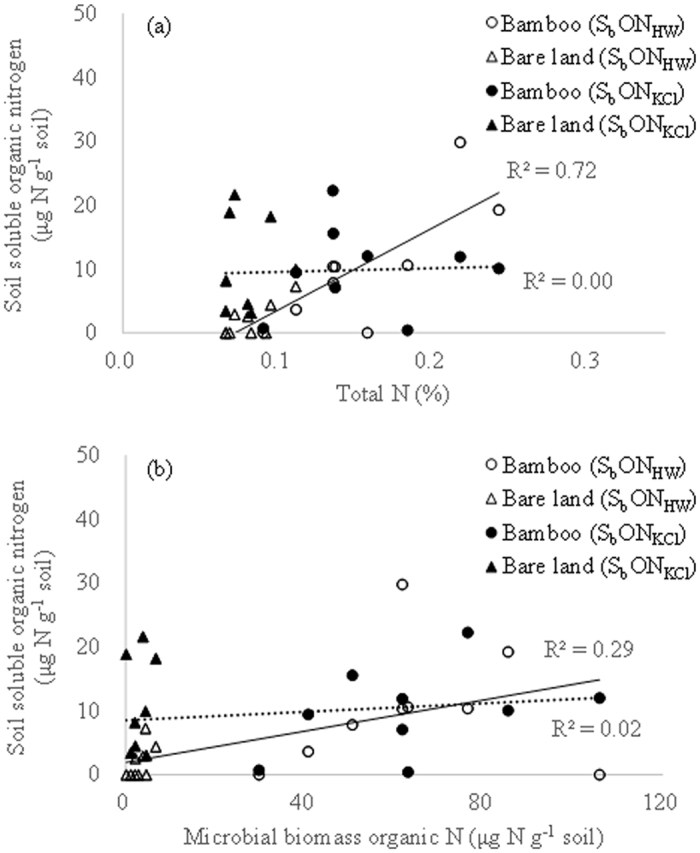
Relationship between soil soluble organic nitrogen in 2 M KCl extracts (S_b_ON_KCl_) and soil total organic nitrogen (**a**) and that between hot water extracts (S_b_ON_HW_) and soil microbial biomass nitrogen (**b**) in thorny bamboo plantations and adjacent bare lands.

The S_b_OC_KCl_ and S_b_OC_HW_ in both bamboo plantations and bare land soils were significantly correlated with TOC and C_mic_, respectively (Fig. 1). However, values for S_b_OC_KCl_ and S_b_OC_HW_ in the bare land soils were clustered and showed less dependence on TOC or C_mic_. In this study S_b_OC_HW_ was more strongly correlated with both TOC and C_mic_ than S_b_OC_KCl_, indicating that S_b_OC_HW_ could be a better reflection of the effects of bamboo on soil TOC and C_mic_ than S_b_OC_KCl_. The overall S_b_ON_HW_ was significantly correlated with both TN and N_mic_ but not with S_b_ON_KCl_ (Fig. 2). The strong correlations between S_b_OC_HW_ and TOC, as well as S_b_ON_HW_ and TN, imply that hot water may help to release more organic substrates from the soils.

The ∆logK (logarithmic ratio of light absorbance of humic acids at 400 and 600 nm; ∆logK = log(A_400_/A_600_)) values observed in soils from thorny bamboo plantations were significantly higher than those of bare land soils (Table 5). The contents of soil acid-hydrolysable C pool I (soil organic C extracted with 5 N H_2_SO_4_; AHPI-C), acid-hydrolysable C pool II (soil organic C extracted with 26 N H_2_SO_4_; AHPII-C), and recalcitrant C pool (residue after acid-hydrolysis; RP-C) were significantly higher in soils from thorny bamboo plantations compared to those of bare land soils. Considering that the TOC contents differed between the two types of soils, the ratios of acid-hydrolysable and recalcitrant contents to TOC were calculated. The ratios of AHPI-C and AHPII-C to TOC (AHPI-C/TOC and AHPII-C/TOC) were higher in bamboo plantation soils, while the ratio of RP-C to total organic C (RP-C/TOC) was higher in bare land soils.

## Discussion

In this study, we compared physicochemical properties of bare land soils and those planted with thorny bamboo. The data from this study shows significantly higher soil C_mic_, N_mic_, respiration rates, total mineralisable N, S_b_OC_HW_/TOC, and C_mic_/TOC in soils from the bamboo plantations compared to bare land soils, mostly due to fast growth and high biomass production of bamboos^10^,^11^. In the present study, thorny bamboo resulted in an increase in TOC content, which also improved the labile SOM (i.e. S_b_OC) of the previously bare lands. For both KCl and hot water extraction methods, S_b_OC was 2–7 times higher in soils from bamboo plantations compared to soils from the adjacent bare lands, indicating the strong contribution of thorny bamboo growth to soil C pools.

Soil organic matter is one of the most important indicators of soil quality^12^, as it improves soil physicochemical properties (e.g. decreasing soil bulk density and increasing soil porosity), and increases soil water content as a result high water holding capacity of SOM^13^,^14^. These benefits provided by SOM may explain the overall improvement of soils at bamboo plantations in the present study, where soil bulk density decreased by ~30% and soil water content increased by 10%. Moreover, the highest TOC in the bamboo plantation from Site 2 helped to improve the soil quality to the extent that Site 2 was observed to have the lowest bulk density and the highest water content of all the sites in the present study.

The higher S_b_OC_HW_/TOC observed in bamboo plantation soils suggested that the observed SOM changes in bamboo plantation soils was not only in the total C pool as a result of litter from the bamboo plants, but also labile fractions as a result of bamboo litter decomposition. The lower S_b_OC_KCl_ and S_b_OC_HW_ of bamboo plantation soils in the current study than those in a previous study conducted in a mountainous area of Central Taiwan^15^ could be due to the fact that soils in the Central Taiwan contained higher TOC. In addition, the considerable lower S_b_OC/TOC from both KCl and hot water extractions of bamboo plantation soils in the current study than those in a mountainous area of Central Taiwan^15^ implys that most of the TOC in badland soils was strongly bound to the soil particles as a result of the high silt and clay contents.

The organic materials and dense roots provided by bamboo have shown to reduce overland flow velocity and infiltration rates^16^,^17^, thereby reducing soil erosion^18^. The export of nutrients associated with sediment loss was found to decrease as a result of bamboo plantations^19^, resulting in an overall accumulation of soil nutrients in bamboo plantations, as well as an improvement in soil quality^20^. The fact that the NH_4_^+^, S_b_ON, and TDN measured in bamboo plantation soils in the current study were distinctively higher in comparison to bare land soils may be due to the less soil erosion in bamboo plantation soils.

The N_mic_ was found to be positively correlated with TN, as soils with higher levels of N support higher microbial biomass^21^. The higher N_mic_/TN ratios at the bamboo plantations in this study indicate that bamboo plantations may have a higher capacity for N retention through the synthesis of N in microbial biomass. Potentially mineralisable N has been considered an active fraction of soil organic N^22^. Thus, the high potentially mineralisable N in the bamboo plantation soils in our study implies that the bamboo plantations contained high levels of active soil organic N.

Labile SOM (S_b_OC and S_b_ON) are the most readily available energy source for soil microbial growth^23^. In the present study, S_b_OC was positively correlated with C_mic_ in bamboo plantation soils. The positive relationships between S_b_OC, TOC, and C_mic_ implies that S_b_OC may be derived from the decomposition of soil TOC as a result of microbial activity^24^. The increase in C_mic_, owing to the readily available energy in S_b_OC, was found to increase the soil respiration rates of bamboo plantation soils. Moreover, higher C_mic_/TOC and S_b_OC_HW_/TOC ratios indicate that the available organic substrates were used more efficiently by microbes in soils planted with bamboos^25^.

The respiration/C_mic_ ratio has been used in microbial studies to indicate the ecological efficiency (i.e. energy required to support the metabolism of per unit C_mic_ in soil) of soil microbial communities^26^,^27^. A high respiration/C_mic_ ratio indicates the inefficient use of energy, while a low respiration/C_mic_ ratio indicates high efficiency, and that higher quantities of C are utilized for biomass production^26^,^28^,^29^. In the present study, soil respiration appeared to be higher in bamboo plantation soils than in bare land soils. However, the respiration/C_mic_ ratio showed a reverse trend, and was significantly lower in soils planted with bamboo than in bare land soils (p < 0.05). This result implies that the lower respiration/C_mic_ ratio of bamboo plantation soils could be attributed to high microbial biomass rather than microbial activity. Moreover, the higher respiration/C_mic_ ratio could be a good indicator that microbes were under greater stress in bare land soils.

The value of ∆logK is a soil index to evaluate the humification status of SOM, and it generally decreases with increasing SOM humification. The lower ∆logK values observed in bare land soils indicate a high degree of humification, as there is little input of fresh litter into the soil. The growth of bamboo adds fresh litter to soils and causes the accumulation of SOM^30^,^31^. Wang, *et al*.^32^ noted that bamboo litter contains a high portion of O-alkyl-C, a C functional group that can be easily decomposed by soil microbes, which is thought to contribute to increases in the labile organic C of soils. The high AHPI-C/TOC and AHPII-C/TOC ratios of soils planted with bamboo support the notion that the decomposition of bamboo litter increases acid-hydrolysable C pools. The acid-hydrolysable C pool is small in size and has a rapid turnover, which responds rapidly to changes in C supply and affects microbial activity^33^,^34^. The higher quantity of acid-hydrolysable C pools in bamboo plantation soils could be one of indicators of their high soil quality. The high RP-C/TOC ratio of bare land soils indicates that the C of the SOM composition is relatively more recalcitrant (i.e. high molecular weight C, irregular structure, and long turnover), and therefore has a higher resistance to chemical degradation and decomposability^35^.

In addition, the fact that the acid-hydrolysable C and S_b_OC displayed similar trends between the sampling sites implies that both methods extracted similar C pools^36^. The observation that acid-hydrolysable C was 10–20 times higher than S_b_OC at all sites may be due to the fact that acid-hydrolysable C contained more slow-turnover C than hot water- or KCl-extracted C^37^. Hot water-extracted soil organic C has been typically considered to be readily metabolisable^38^, while acid-hydrolysable C has been shown to be composed of bioreactive C in soil, even if it is not readily used by microbes^39^. Therefore, acid-hydrolysable C is less sensitive to environmental changes, while both acid-hydrolysable C and S_b_OC represent the labile fractions of soil C pools^36^.

In conclusion, this study demonstrates that planting of thorny bamboos in uninhabitable badland soils appears to be a successful practice for soil amelioration. The thorny bamboo helped to increase SOM and improve soil physiochemical properties that contribute to soil improvement.

## Methods

### Site and soil sampling

**Figure 3:**
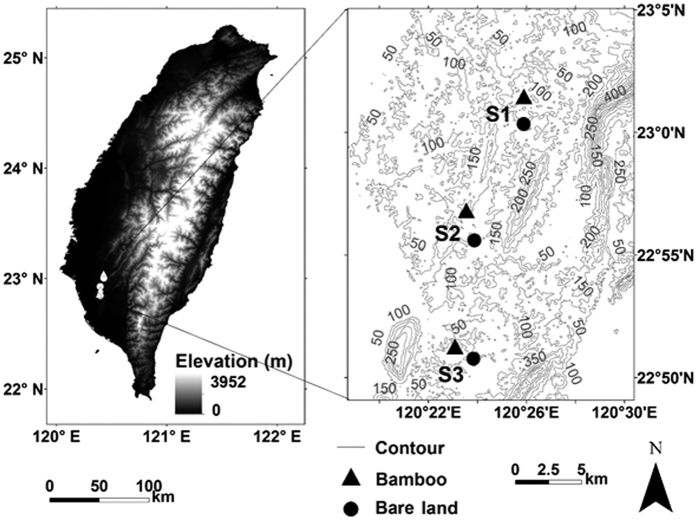
Map of soil sampling locations in Southern Taiwan. Elevation data used to create Fig. 3 was obtained using the Advanced Spaceborne Thermal Emission and Reflection Radiometer (ASTER) digital elevation model (DEM) at a resolution of 30 m ( https://asterweb.jpl.nasa.gov/gdem.asp), while contour lines were created using the ArcGIS v.10.0 software package (ESRI [ http://www.esri.com/], Redlands, CA, USA). (Triangles: thorny bamboo sites; circles: bare land sites).

This study was carried out at three locations in southwestern Taiwan—Site 1 at Zuozhen, Tainan (120°26′E, 23°0′N), Site 2 at Longqi, Tainan (120°23′ E, 22°54′N), and Site 3 at Tianliao, Kaohsiung (120°24′E, 25°51′N)—with an average altitude of 100–300 m (Fig. 3).

The lands chosen as sampling locations for this study are owned by the Forestry Bureau and accessed for public. Research proposals were approved and granted by the Ministry of Science and Technology, Taiwan. Field and laboratory studies did not involve any animal husbandry, nor any protected or endangered biological species.

**Figure 4:**
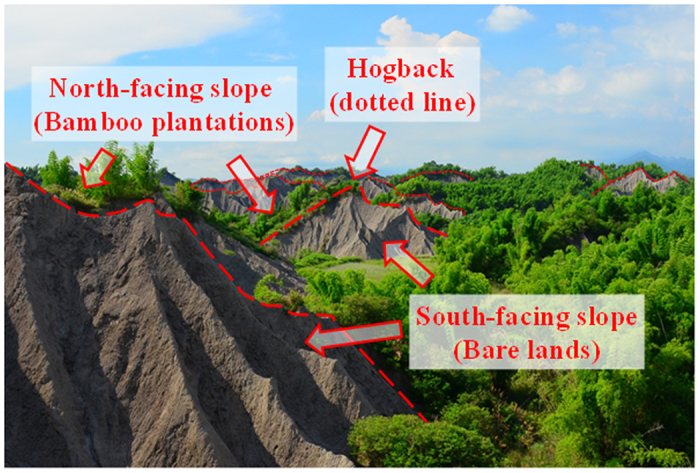
Landscape of mudstone badlands at Zuozhen in Tainan City, Southern Taiwan. South-facing slopes are barren while north-facing slopes are covered with thorny bamboo (photo by CYC, corresponding author).

At each site, it involved badland soils with and without bamboos. In badland, desiccation cracks occur on the slope surfaces extending to a few centimeters in the dry season. During the rainy season, the soil expands and closes the cracks to form crusts in 1–2 cm thick^3^. Soils in the three studied locations have high clay and silt contents (clay: 27–44%; slit: 31–47%), primarily inherited from the parent mudrock. Based on U.S. Soil Taxonomy^40^, soils in the badland lacking of vegetation were classified as Typic Eutrustepts, and those with the bamboo as Typic Dystrudepts. Thorny bamboo, which was cultivated in the early 1900s^41^ by local residents for bamboo shoots and stems, is the predominant plant on the north-facing sides of hogbacks (ridges with a sharp summit and steeply sloping sides) (Fig. 4). The south-facing sides of hogbacks are composed of bare land soils with no plant coverage. Interestingly, the downstream valley near these badlands (only a few km away) are well cultivated as orchards with various fruit trees, such as mango, jujube, and banana, indicating that original mudstone soils can be ameliorated for agricultural uses after certain improvements. At each site, three transects (replications), 50 m apart, were demarcated at both north-facing and south-facing sides of the hogbacks. Thus, there were a total of 18 transects (composited samples) for the study (2 vegetation types: bamboo and bare land soil × 3 replications × 3 locations for each soil type). A soil sample, composited of 8 soil cores, was collected in each transect by a soil auger (4.25 cm in diameter) and stored in plastic bag. The collected soil samples were stored at 4 °C until laboratory analysis.

### Laboratory analyses

#### General properties of soil

Soil pH was measured in a 1:1 soil to water suspension using a portable Jenco 6009 pH/mV meter. Soil electrical conductivity was measured using a saturated extract method^42^. Soil total organic carbon (TOC) was analysed using a Fisons NA1500 elemental analyser (ThermoQuest Italia, Milan, Italy) after removing carbonate with 1 N HCl. Briefly, 0.3 g of soil was weighted out, mixed with 0.5 ml of HCl and air dried for 12 h before analysing with the elemental analyser. Soil TN was determined using the Kjeldahl digestion method^43^. Deionized water was added to the digested solution to a volume of 100 ml and analysed using the previously described indophenol method^44^ and measured spectrophotometrically (UV-1201, Shimadzu Corp., Kyoto, Japan). To determine the soil water content, soil was oven dried at 105 °C for 24 h and weighed for water loss. Soil texture was determined using the hydrometer method^45^. Soil bulk density was determined using the weight and volume of soil samples collected via cores.

#### Soil extractable nutrients

Soil nutrient contents, including NH_4_^+^, NO_3_^−^, S_b_ON, and S_b_OC, were measured using two different extraction methods, KCl extraction and hot water extraction, described in Chen *et al*.^46^ with modification. For the KCl extraction, 5 g of air-dried soil from each replicate sample was weighted out, placed in a 250-ml conical flask, and injected with 50 ml of 2 M KCl. The flask was sealed with plastic paraffin film and shaken for 60 min at 150 rpm. The solution was collected by filtering the slurry using filter paper (Whatman No. 5).

For the hot water extraction method, 6 g of air-dried soil samples were weighed, placed in a 50-ml centrifuge bottle, and injected with 30 ml of distilled deionized water. The bottle was placed in an 80 °C water bath and incubated for 18 h, subsequently shaken for 5 min at 150 rpm, and the solution was collected by filtering the slurry using the same size filter paper as KCl extracts. Extracted NH_4_^+^ was analysed using the indophenol method with a spectrophotometer at 635 nm wavelength (UV-1201, Shimadzu Corp., Kyoto, Japan). The NO_3_^−^ was determined using the cadmium reduction method^47^ with a flow injection analyser (SP-8001, Metertech Inc., Taipei City, Taiwan). Soil TDN was digested to NO_3_^−^ using the persulfate method^48^ and was then analysed as aforementioned with a flow injection analyser. The S_b_ON was calculated by subtracting NO_3_^−^ and NH_4_^+^ from TDN. The S_b_OC was measured using a TOC analyser (1010, O.I. Analytical, Texas, USA).

#### Soil microbial biomass C and N

Soil C_mic_ and N_mic_ were analysed using a chloroform-fumigated extraction method^49^. Briefly, four 10 g aliquots of soil were weighed out from each sample and placed in a 250-ml conical flask. Then, soil samples were equally separated into two sets of 36 samples. One set of soil samples were fumigated using chloroform for 24 hours and extracted with 50 ml of potassium sulphate (0.5 M K_2_SO_4_). The other set of soil samples were directly extracted with 50 ml of K_2_SO_4_ (0.5 M). The C in K_2_SO_4_ solutions was determined using a TOC analyser and N was analysed using a previously described protocol^50^. The C_mic_ and N_mic_ were calculated as the differences between the fumigated and unfumigated K_2_SO_4_-extractable C and N by multiplying coefficients (K_EC_ = 2.22 and K_EN_ = 4.95) [(fumigated C (N) − unfumigated C (N)) × coefficients]^51^,^52^.

#### Soil respiration

Soil respiration rate was estimated from the total amount of CO_2_-C during a 3-day incubation using an alkali method^15^,^53^. Briefly, 20 ml of 0.05 M NaOH was injected into a 250-ml flask and a plastic tube and 20 g of fresh soil sample was placed in the flask. After 3 days of incubation at 25 °C, the 20 ml NaOH solution was titrated using 0.05 M HCl with phenolphthalein and BaCl_2_.

#### Total mineralisable N

Total mineralisable N was determined using the waterlogging incubation method^54^. Briefly, 5 g of moist field soil was weighed out from each sample, placed in a 250-ml centrifuge bottle with 25 ml of distilled deionized water, and incubated and shaken in an incubator at 40 °C for 7 d. After incubation, 25 ml of 4 M KCl was injected into the centrifuge bottle and shaken for 1 h at 150 rpm. Subsequently, the bottle was centrifuged at 2000 rpm for 20 min and 50 ml of water was extracted and filtered for the determination of NH_4_^+^ concentration.

#### Extraction of humic acids for photometric analysis

One gram of air-dried soil from each sample was weighed and shaken at 150 rpm with 30 ml of 0.1 N NaOH at 100 °C for 30 min. The Na_2_SO_4_ (2 ml) was added and the samples were subsequently centrifuged at 10,000 × *g* for 15 min. Precipitates were further extracted with a mixed solution of 20 ml of 0.1 N NaOH and 3% Na_2_SO_4_, and centrifuged at 10,000 × *g* for 15 min, twice. The extractant was quantified to 100 ml with deionized water and then acidified with 1 ml concentrated H_2_SO_4_ (98%). Precipitates were subsequently dissolved in 30 ml of 0.01 N NaOH to determine the ratio of humic acids^9^ using a spectrophotometer (Hitachi U-2000). The light absorbance of humic acids was measured at 400 and 600 nm to determine their molecular fractions, as the smaller fractions of humic substrates tend to absorb shorter wavelengths of light^55^. The degree of humification was determined by calculating the ∆logK value, which is the logarithmic ratio of the absorbance at 400 and 600 nm (∆logK = log(A_400_/A_600_))^56^. The ∆logK is an inverse index of the condensation of the aromatic network in the macromolecules of humic acids, representing the degree of humification.

#### Determination of acid-hydrolysable and recalcitrant C

Acid hydrolysis is a chemical fractionation method used to separate acid-hydrolysable SOM and recalcitrant SOM pools (unhydrolysable)^33^. The two-step acid hydrolysis is used to isolate and quantify labile and recalcitrant substances of SOM with sulphuric acid (H_2_SO_4_) as the extractant^57^. Briefly, 0.5 g of each soil sample was hydrolysed with 20 ml of 5 N H_2_SO_4_ at 105 °C for 30 min in Pyrex flasks with Allihn condensers. Samples were centrifuged at 20,000 × *g* for 10 min and decanted to collect the hydrolysable SOM from each sample. Residues were flushed with 20 ml of deionized water, and the extract was added to the previous hydrolysate. The C from the hydrolysate was regarded as the AHPI-C. The remaining residual SOM was hydrolysed with 2 ml of 26 N H_2_SO_4_ overnight while shaking continuously at room temperature. The concentration of H_2_SO_4_ in the sample was brought down to 2 N by dilution and hydrolysed for 3 h by heating at 105 °C. The resulting hydrolysate was considered as AHPII-C. The remaining SOM in the sample, regarded as RP-C, was flushed with 30 ml of deionized water and dried at 60 °C in a pre-weighed crucible. AHPI-C and AHPII-C were measured using a TOC analyser (1010, O.I. Analytical, Texas), while the RP-C was determined by an NSC elemental analyser (Fisons NA1500).

All analyses in the study were performed in duplicate. All results have been converted on dry weight basis.

### Statistical analyses

Differences in soil nutrients between bare lands and bamboo plantations at all locations were tested for significance using a one-way analysis of variance (one-way ANOVA). When the one-way ANOVA revealed interactions between sites and soil physicochemical properties, Tukey’s honestly significant difference (HSD) test was applied to further test the means of all pairs of dependent variables. Relationships between two soil properties were analysed using bivariate regression analysis. All statistical analyses were performed using JMP (Version Pro 10, SAS Institute, Cary, NC, USA). The level of significance was set at 0.05 for all tests.

## Additional Information

**How to cite this article**: Shiau, Y.-J. *et al*. Improvement in the biochemical and chemical properties of badland soils by thorny bamboo. *Sci. Rep.* **7**, 40561; doi: 10.1038/srep40561 (2017).

**Publisher's note:** Springer Nature remains neutral with regard to jurisdictional claims in published maps and institutional affiliations.

## Figures and Tables

**Table 1:** Soil properties (0–10 cm) under three thorny bamboo plantations and adjacent bare lands in Southern Taiwan.

Site	Vegetation	pH	EC^1^ (mS/cm)	Water Content (%)	Clay (%)	Sand (%)	Silt (%)	Texture	Bulk density	Total organic C (%)	Total N (%)	Classification
1	Bare land	8.63^a^	7.59^b^	7.2^d^	43.5^a^	20.2^c^	36.3^a^	clay	1.55^a^	0.34^c^	0.07^d^	Typic Eutrustepts
	Bamboo	8.20^ab^	0.80^c^	15.9^b^	30.2^ab^	39.1^a^	30.7^a^	clay loam	1.23^b^	0.90^b^	0.13^bc^	Typic Dystrudepts
2	Bare land	8.42^ab^	12.59^a^	8.7 ^cd^	28.9^b^	26.7^bc^	44.4^a^	clay loam	1.81^a^	0.45^c^	0.08^d^	Typic Eutrustepts
	Bamboo	6.15^c^	0.66^c^	20.5^a^	29.5^b^	36.3^ab^	34.2^a^	clay loam	1.11^b^	1.77^a^	0.21^a^	Typic Dystrudepts
3	Bare land	8.33^ab^	13.33^a^	10.0^c^	27.1^b^	25.5^bc^	47.4^a^	clay loam	1.80^a^	0.47^c^	0.10 ^cd^	Typic Eutrustepts
	Bamboo	8.17^b^	0.65^c^	17.0^b^	29.0^b^	26.8^bc^	44.2^a^	clay loam	1.23^b^	1.11^b^	0.14^b^	Typic Dystrudepts

^1^EC = Electrical Conductivity. Values with the same superscripted letters in each column are not significantly different at *p* = 0.05 based on the Tukey’s HSD comparison.

**Table 2:** Concentrations of soluble N and soluble organic C in 2 M KCl extracts, S_b_ON/TN ratio, and S_b_OC/TOC ratio in the top 10 cm of soil from thorny bamboo plantations and adjacent bare land in Southern Taiwan.

Site	Vegetation	S_b_OC_KCl_ (μg/g soil)	NH_4_^+^_KCl_ (μg/g soil)	NO_3_^−^_KCl_ (μg/g soil)	S_b_ON_KCl_ (μg/g soil)	TDN_KCl_ (μg/g soil)	S_b_OC_KCl_/TOC (%)	S_b_ON_KCl_/TN (%)
1	Bare land	27.8^c^	4.4^bc^	3.9^b^	5.4^a^	13.7^c^	0.84^a^	0.79^a^
	Bamboo	41.9^b^	7.5^bc^	4.4^b^	3.5^a^	15.4^c^	0.49^bc^	0.32^a^
2	Bare land	26.5^c^	4.0^c^	8.5^ab^	12.2^a^	24.7^bc^	0.60^b^	1.81^a^
	Bamboo	58.0^a^	20.6^a^	15.0^a^	11.3^a^	46.9^a^	0.33^c^	0.58^a^
3	Bare land	27.4^c^	6.5^bc^	6.4^b^	10.4^a^	23.3^bc^	0.59^b^	1.08^a^
	Bamboo	41.8^b^	10.9^b^	4.9^b^	15.0^a^	30.8^b^	0.38^c^	1.29^a^

[Soil soluble organic carbon (S_b_OC); ammonium (NH_4_^+^); nitrate (NO_3_^−^); Soil soluble organic nitrogen (S_b_ON); total dissolved nitrogen (TDN); total organic carbon (TOC); total nitrogen (TN)]. Values with the same superscripted letters in each column are not significantly different at *p* = 0.05 based on the Tukey’s HSD comparison.

**Table 3:** Concentrations of soluble N and soluble organic C in hot water extracts, S_b_ON/TN ratio, and S_b_OC/TOC ratio in the top 10 cm of soil from thorny bamboo plantations and adjacent bare land in Southern Taiwan.

Site	Vegetation	S_b_OC_HW_ (μg/g soil)	NH_4_^+^_HW_ (μg/g soil)	NO_3_^−^_HW_ (μg/g soil)	S_b_ON_HW_ (μg/g soil)	TDN_HW_ (μg/g soil)	S_b_OC_HW_/TOC (%)	S_b_ON_HW_/TN (%)
1	Bare land	62.8^c^	1.5^c^	2.3^b^	0.9^b^	3.8^d^	1.88^c^	0.11^a^
	Bamboo	215.8^b^	9.4^b^	2.8^ab^	4.7^b^	16.9^bc^	2.40^b^	0.43^a^
2	Bare land	70.3^c^	1.9^c^	4.4^ab^	1.0^b^	6.6 ^cd^	1.58^c^	0.22^a^
	Bamboo	543.7^a^	16.4^a^	13.4^a^	16.4^a^	41.5^a^	3.01^a^	0.72^a^
3	Bare land	78.6^c^	2.5^c^	4.1^ab^	3.9^b^	9.9 ^cd^	1.69^d^	0.37^a^
	Bamboo	282.8^b^	14.1^ab^	3.7^ab^	9.5^ab^	27.3^b^	2.54^b^	0.74^a^

[Soil soluble organic carbon (S_b_OC); ammonium (NH_4_^+^); nitrate (NO_3_^−^); Soil soluble organic nitrogen (S_b_ON); total dissolved nitrogen (TDN); total organic carbon (TOC); total nitrogen (TN)]. Values with the same superscripted letters in each column are not significantly different at *p* = 0.05 based on the Tukey’s HSD comparison.

**Table 4:** Soil microbial biomass, potentially mineralisable nitrogen, and microbial quotient in soils from thorny bamboo plantations and adjacent bare lands in Southern Taiwan.

Site	Vegetation	C_mic_ (μg C/g soil)	Respiration rate (μg C/g soil/hr)	N_mic_ (μg N/g soil)	Mineralisable N (μg N/g soil/d)	C_mic_/TOC (%)	N_mic_/TN (%)	Respiration/C_mic_ (g CO_2_-C/g microbial-C/h)
1	Bare land	4.3^c^	1.33^c^	1.8^d^	0.00^c^	0.13^b^	0.26^b^	0.22^a^
	Bamboo	40.0^b^	3.13^ab^	44.5^c^	0.87^b^	0.51^a^	3.46^a^	0.11^b^
2	Bare land	3.5^c^	0.88^c^	2.2^d^	0.22^bc^	0.08^b^	0.28^b^	0.25^a^
	Bamboo	69.0^a^	3.73^a^	84.4^a^	3.04^a^	0.40^a^	4.37^a^	0.05^b^
3	Bare land	5.8^c^	1.28^c^	5.2^d^	0.00^c^	0.13^b^	0.56^b^	0.31^a^
	Bamboo	35.9^b^	3.79^b^	63.0^b^	0.79^bc^	0.33^a^	4.89^a^	0.08^b^

[Microbial biomass carbon (C_mic_); microbial biomass nitrogen (N_mic_); total organic carbon (TOC); total nitrogen (TN)]. Values carrying the same letters in each column are not significantly different at p = 0.05 based on the Tukey’s HSD comparison.

**Table 5:** The degree of humification as indicated by ∆logK, acid-hydrolysable pool I (AHPI-C) and II carbon (AHPII-C), recalcitrant pool carbon (RP-C), and ratios of labile and recalcitrant carbon to total organic carbon (TOC) of soils from three thorny bamboo plantations and adjacent bare lands in Southern Taiwan.

Site	Vegetation	∆logK	AHPI-C (mg C/g soil)	AHPII-C (mg C/g soil)	RP-C (mg C/g soil)	AHPI-C/TOC	AHPII-C/TOC	RP-C/TOC
1	Bare land	0.35^b^	0.31^c^	0.30^c^	5.70^bc^	0.04^c^	0.04^d^	0.75^a^
	Bamboo	0.85^a^	3.79^b^	2.10^b^	8.88^ab^	0.28^b^	0.16^ab^	0.68^ab^
2	Bare land	0.48^b^	0.27^c^	0.60^c^	5.31^bc^	0.04^c^	0.09 ^cd^	0.76^a^
	Bamboo	0.73^a^	7.72^a^	3.66^a^	11.05^a^	0.42^a^	0.20^a^	0.59^b^
3	Bare land	0.37^b^	0.18^c^	0.32^c^	4.74^c^	0.03^c^	0.05^d^	0.68^ab^
	Bamboo	0.73^a^	3.20^b^	1.52^bc^	8.01^abc^	0.24^b^	0.12^bc^	0.65^ab^

Values carrying the same letters in each column are not significantly different at *p* = 0.05 based on the Tukey’s HSD comparison.
